# Recent Advances in Intraoperative Brainstem Auditory Evoked Potential Monitoring during Microvascular Decompression Surgery for Hemifacial Spasm

**DOI:** 10.3390/life13091789

**Published:** 2023-08-22

**Authors:** Sang-Ku Park, Hyun Seok Lee, Kyung Rae Cho, Kwan Park

**Affiliations:** 1 Department of Neurosurgery, Konkuk University Medical Center, Seoul 05030, Republic of Korea20220205@kuh.ac.kr (H.S.L.);; 2 Department of Neurosurgery, School of Medicine, Sungkyunkwan University, Seoul 03063, Republic of Korea

**Keywords:** brainstem auditory evoked potentials, microvascular decompression, hemifacial spasm, vestibulocochlear nerve damage

## Abstract

Brainstem auditory evoked potential (BAEP) testing during microvascular decompression (MVD) is very important in the treatment of hemifacial spasm (HFS). The reason for this is that the vestibulocochlear nerve is located immediately next to the facial nerve, so the vestibulocochlear nerve may be affected by manipulation during surgery. BAEP testing for detecting vestibulocochlear nerve damage has been further developed for use during surgery. In most HFS patients with normal vestibulocochlear nerves, the degree of vestibulocochlear nerve damage caused by surgery is well-reflected in the BAEP test waveforms. Therefore, real-time testing is the best way to minimize damage to the vestibulocochlear nerve. The purpose of this study was to review the most recently published BAEP test waveforms that were obtained during MVD surgery to determine the relationship between vestibulocochlear nerve damage and BAEP waveforms.

## 1. Introduction

Due to the acquisition of BAEP waveforms with click sound stimulation in the human head for the first time in the 1980s [1] and the transition of equipment software from analog systems to digital systems, more sophisticated BAEP waveforms have been obtained [2]. Because of Moller AR and Jannetta PJ, we can now test BAEPs during surgery [3,4]. Several studies have been published since the introduction of BAEP testing.

In 2004, M P Sindou highlighted the importance of intraoperative neurophysiological monitoring and, in particular, analyzed in detail the change in wave V latency in the BAEP waveform. In this prospective study of 84 consecutive patients, hearing loss due to intraoperative changes in brainstem auditory evoked potentials and their corresponding warning values were prevented. If the latency of wave V was delayed by 0.4 ms, it was a “watching” signal; if it was delayed by 0.6 ms, it was a “warning” of real risk and a “critical” warning if it was extended by 1.0 ms, where it was then classified into each stage as a “critical” warning [5]. He described the meaning of wave I loss as well as wave V loss when hearing loss occurred after surgery [6].

Hatayama attributed postoperative hearing loss to changes in amplitude rather than the extended latency of wave V. All patients who showed a decrease in amplitude of more than 40% of the wave V had prolonged latency, which was reported to be highly related to postoperative hearing loss [7].

In slight contrast to the above, various published studies have mentioned that amplitude is more important than latency. These papers are associated with the latency of wave V and hearing loss, but a reduction in amplitude of more than 50% was highly related to hearing loss [8].

The reason why there are various warning criteria is that each surgeon interprets postoperative results differently because each surgeon administers the BAEP test differently. According to the American Clinical Neurophysiology Society, a V latency longer than 1 ms or a 10% increase in latency and/or a 50% reduction in amplitude is a warning criterion [9]. For the BAEP test, it is suggested that the stimulation rate be 8–10 Hz and the average number of tests be 1000–4000 [10].

Despite the clear proposals to test BAEPs as described above, in some hospitals, the stimulation rate is 31.1 Hz and the average number of tests is 1000–4000 [11]. Another hospital has reported a stimulation rate of 17.5 Hz and an average number of 256 (at least) tests [12]. This reason may be that a test method was learned to reduce hearing loss from many surgical experiences.

Previously, the incidence of postoperative hearing loss has been reported to range between 7.7 and 20% when surgery was performed without intraoperative BAEP monitoring [5]. Currently, with intraoperative BAEP monitoring, the average postoperative hearing loss incidence rate is 3.4% [13].

In this paper, we present findings on the meaning of BAEP waveform changes and the latest test of a method that can further reduce the incidence of postoperative hearing loss by monitoring BAEPs during surgery.

## 2. Methods

In the BAEP test, it is possible to analyze more functions of the cochlear nerve in detail than when using the real-time method rather than the conventional method. Therefore, in this study, we will focus on studies conducted with the real-time BAEP method.

### 2.1. BAEP Test in Routine

Short-latency auditory evoked potentials use click sound stimulation to maximize activation from the vestibulocochlear nerve to the brainstem; the units of stimulation are dB nHL (normal hearing level) or dB SPL (sound pressure level).

The stimulus intensity should be set at a level to produce clear BAEPs but not cause tympanum damage. In the case of testing with SPL, the threshold is first measured. The intensity is increased sequentially from 0 dB and the test is conducted with a value obtained by adding 60 dB to the intensity at which the sound begins to be heard. A maximum click intensity of 120 dB SPL or 90 dB nHL is commonly utilized. We do not test at higher than these levels of intensity. To mask crossover responses, white noise at 60 dB SPL or 30–35 dB HL is applied to the contralateral ear. Clicks can be of two polarities, either condensation or rarefaction, depending on the initial movement of the diaphragm of the transducer.

We attempt to obtain a pure waveform by removing artifacts from the patient under examination using an 8–10 Hz stimulation rate, where the amplitude of the waveform is observed to be the largest and the average number of tests is 1000–4000 times, which is rather lengthy [10].

To explain in more detail, the reason for testing at 10 Hz is that when testing with a faster stimulation rate of 30 Hz or 70 Hz, the amplitude of wave V is small and the interpeak latency from wave I to wave V is further extended, that is, short-latency auditory evoked potentials, which measure the function of a person’s vestibulocochlear nerve to the brainstem, are the best waveforms when tested at 10 Hz [14]. In particular, movement or coughing during the test affects the averaging of the BAEP waveform, so at least 1000 tests are required to acquire an accurate average to exclude these effects.

### 2.2. BAEP Testing in Intraoperative Neurophysiological Monitoring (INM)

#### 2.2.1. Real-Time BAEPs

##### Stimulation Rate and Averaging Time

Generally, in an awake state, these impure waveforms do not affect the average (the average being at least 1000 tests, with each test lasting for at least 100 s until the waveform is stable to obtain a waveform with good reproducibility as the patient’s unique waveform is needed). Since the BAEP test performed during surgery is performed under general anesthesia, there are no artifacts due to the patient’s minute movements during the BAEP test. Therefore, a very pure waveform is obtained. Stimulation rates up to 100 are possible with most medical devices. In other words, it is possible to test BAEPs that can enter 100 stimuli per second.

If the BAEP test is performed intraoperatively in the same way as the routine test, a stimulation rate of 10 Hz for an average of 1000 trials requires approximately 100 s to obtain the BAEPs. This relative increase in duration may limit the prevention of postoperative hearing loss with the development of an INM machine with a high signal-to-noise ratio and the disintegration of the waveform amplitude that occurs with a higher stimulation rate is significantly improved. According to this study, no significant difference was found in the BAEP waves obtained by measuring the stimulation rate faster than 40 Hz and measuring it at 10 Hz; it is possible to obtain a reliable waveform with fewer trials. Reliable waveforms have been detected at 43.9 Hz/s in an average of 400 trials [15].

Therefore, it is possible to obtain BAEPs that test within 10 s with a combination of an averaging time of 400 and a stimulation rate of 40–50 Hz. In this study, these test methods are called real-time BAEPs.

**Figure 1:**
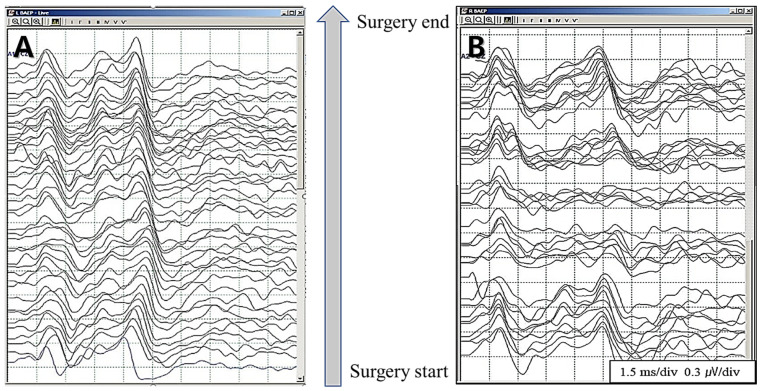
Without any special abnormality, latency is observed to be prolonged slowly and steadily (**A**). In some cases, even if the waveform is lost, it can be recovered by taking quick action (**B**).

By checking the results of BAEPs within 10 s, the continuous wave V latency delay state can be observed in great detail (Figure 1A) and, even if wave V loss occurs, the point of time can quickly be identified; this can help greatly in restoring the waveform (Figure 1B).

#### 2.2.2. Warning Criteria

When analyzing the relationship between the change in the BAEP waveform tested by the real-time BAEP method during surgery and hearing loss after surgery, we could apply a known V latency longer than 1 ms or a 10% increase in latency and/or with a 50% reduction in amplitude as a warning criterion.

However, it was possible to distinguish the change in wave V in detail. First, it was found that when a weak influence from surgery occurred, a change in latency occurred and, when it was affected more strongly, a change in amplitude occurred. The latency of wave V was gradually extended to 1 ms continuously, while the amplitude rapidly decreased by 50% within 10 s. Therefore, in most cases, the change in latency occurred first, and then the change in amplitude occurred later. In some cases, if a serious effect was received within a few seconds, latency was not prolonged, the amplitude of wave V suddenly decreased by more than 50%, and loss occurred [16].

A study observed changes in BAEP waveforms tested using the real-time method during surgery on 606 patients who visited the hospital for hemifacial spasm and underwent MVD surgery. In this study, the patients were divided into six subdivided groups [17]. Looking at Group 4, when latency was prolonged by 2 ms and amplitude was reduced by more than 50%, 36 patients accounted for 5.9% of the total and 1 patient had postoperative deafness. In Group 5, amplitude loss occurred and did not recover in 5 patients (0.83% of the total) and all of them were deaf after surgery. In Group 6, amplitude loss occurred and recovered; there were 23 patients with transient loss, accounting for 3.8% of the total; there were no total deaf patients after surgery.

Therefore, it could be seen that the permanent loss of wave V in the BAEP wave was closely related to postoperative hearing loss. Additionally, when latency 2 ms prolongation occurred, a decrease in amplitude often occurred and, when latency 2 ms prolongation occurred, loss of amplitude occurred suddenly in some cases. Therefore, if latency is extended by 2 ms, you should be aware that it can change to a very serious state (Table 1).

In the real-time BAEP tests, latency changes were always observed first, followed by amplitude changes. In addition, a latency change was observed slowly and continuously and the amplitude change suddenly decreased by more than 50% within 10 s. When BAEPs were tested quickly in real time, it could sometimes be observed that wave V latency was extended up to 3 ms. With such a quick BAEP test, vestibulocochlear nerve damage was quickly reflected in the BAEP waveform, so we could minimize vestibulocochlear nerve damage with surgical manipulation. As a result, much transient loss tended to be observed in the real-time BAEP method and, in most cases of transient loss, hearing was normal after surgery.

**Figure 2:**
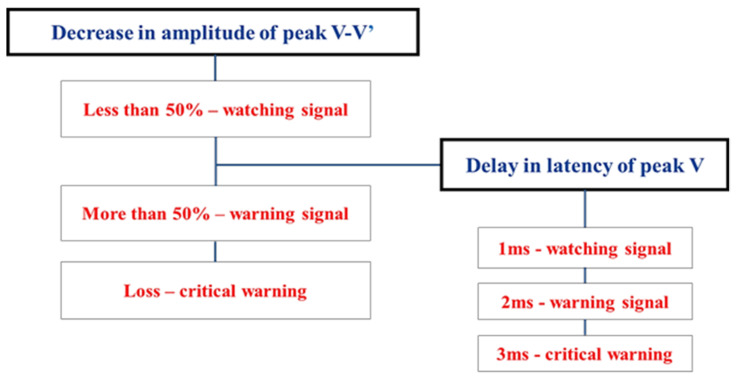
Correlation of amplitude and latency in warning criteria when using real-time BAEPs (data from [17]).

The relationship between the amplitude and latency of BAEP wave V is summarized in the table below (Figure 2).

##### Prewarning Sign

**Figure 3:**
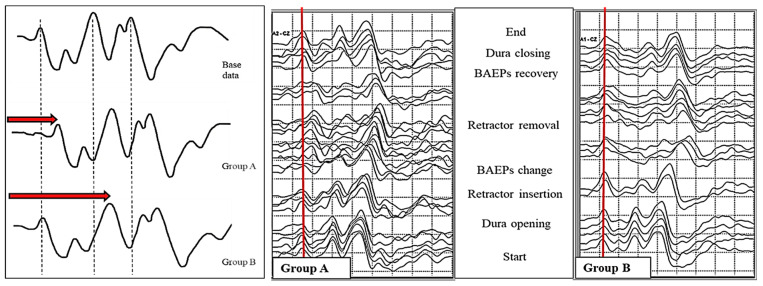
Differences in consecutive brainstem auditory evoked potentials between the study groups.

Until now, only absolute latency has been observed when measuring the change in the latency of BAEP wave V. However, when the BAEP wave V latency was extended beyond 1 ms, it was classified into two patterns. This was the case when the wave V latency was extended in wave I (Group A) and when the wave V latency was extended in wave III (Group B). During surgery, the cochlear nerve was stretched under the influence of surgical manipulation. The reason Gr A occurred was that, as the nerves stretched in the direction of the brainstem, the cochlear part was affected, causing expansion in wave I. The reason why Gr B occurred was thought to be that the area around the brainstem was directly affected and then expansion occurred in wave III [18] (Figure 3).

We analyzed the maximal change in wave V after observing the prewarning changes in BAEPs and found significant differences between the two groups. Analyzing the percentage within each group, the case where the wave V latency was extended by more than 1 ms was observed more in Gr B, with 34.6% in Gr A and 50.6% in Gr B; however, transient loss was observed more in Gr A, with 29.6% for Gr A and 20.0% for Gr B.

We also analyzed the final change in wave V after observing the prewarning changes in BAEPs and found significant differences between the two groups here, too. When the intraoperative BAEP wave V latency was extended by more than 1 ms, 88.9% of patients recovered to less than 1 ms before the end of surgery in Gr A; in 9.9% of patients, the operation was completed with a condition that was longer than 1 ms. In Gr B, 65.6% of patients recovered well within 1 ms immediately before the end of surgery; in 28.1% of patients, the operation was terminated with a prolongation of more than 1 ms. In particular, BAEP wave loss was observed in 1 patient (1.2%) in Gr A and in 10 patients (6.3%) in Gr B.

The two patterns showed different postoperative patterns. In wave I, there was no postoperative hearing loss in patients with both temporary and permanent loss of BAEP in the case of dilation. However, when expansion occurred in wave III, 3 out of 10 patients with permanent loss of BAEPs developed total hearing loss and 2 out of 32 patients with temporary loss of BAEPs developed partial hearing loss.

In summary, if the BAEP wave V latency was prolonged by more than 1 ms, the recovery of the waveform could be better when prolonged (Gr A) occurred in wave I than when prolonged (Gr B) occurred in wave III. When prolonged (Gr B) occurred in wave III, it was a serious situation in which there was considerable waveform loss; hearing loss after surgery also increased (Table 2).

Previously, a study evaluated the changes in the amplitude and latency of BAEP wave III during MVD and the association with postoperative HL. According to this study, when hearing loss was evaluated after surgery in cases where wave III was maintained and only wave V was lost, or when both wave III and wave V were lost, it was found that the amplitude change in wave III had a greater variation in all the steps of surgery [19]. The conclusions of this study and the prewarning sign study are considered to be the same.

##### Significance of Wave I

Loss of BAEP waveforms is closely related to postoperative hearing loss. The change in the BAEP waveform in the main procedure during surgery is reflected in the waveform immediately when the cochlear nerve is damaged due to traumatic mechanical damage. In addition, since it takes time to affect the cochlear nerve due to poor blood circulation, it takes some time for the effects of vascular circulation damage to be reflected in BAEPs [20]. For this reason, changes in the BAEP waveform can be observed very quickly in the case of traumatic mechanical damage. However, in the case of vascular circulation damage, changes may occur after 10 min.

More importantly, in the case of traumatic mechanical damage, the existing warning criteria of a 1 ms delay and a 50% reduction in wave V are applied. In the case of vascular circulation damage, the waveform disappears suddenly and without warning signs.

When traumatic mechanical damage is severely affected, BAEP wave loss occurs, but wave I is always present. Wave I is present in the BAEP waveform of patients with hearing disturbance caused by brain tumors; latency delayed or decreased in amplitude of the remaining waveforms, wave III or V, is similar to that observed.

**Figure 4:**
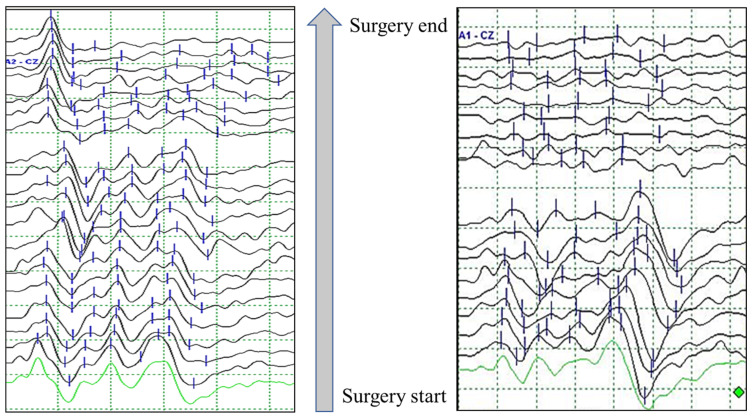
Example of brainstem auditory evoked potentials (BAEPs) according to wave I persistence/absence in patients with wave V loss during microvascular decompression surgery for hemifacial spasm.

That is, the presence of wave I in the BAEP waveform means that the sound transmission ability to the cochlea is normal and that there is a problem with the process from the cochlear nerve to the brainstem. In fact, when looking at the postoperative hearing status of patients in which wave I is present and all other waveforms are lost, most cases show only partial hearing loss (Figure 4).

However, in the case of vascular circulation damage, a waveform without wave V is suddenly observed without extension of or decrease in wave V and all waveforms are lost without even wave I. That is, the vestibular cochlear system is affected as a whole. In this way, patients who have observed BAEP wave loss who have completed surgery but not recovered have very serious and diverse symptoms after surgery. HL is naturally accompanied by tinnitus, dizziness, and hoarseness (Table 3).

It is very important that whether wave I exists when BAEP wave loss occurs is observed and that the BAEP test is not neglected just because the main procedure has been completed during surgery. Testing must continue until the end of surgery.

In a previous study, a wave I amplitude decrease was considered a warning signal of cochlear dysfunction. It was hypothesized that AICA and labyrinth arterial flow reduction were associated due to the manipulation of these vessels [6]. The conclusions of this study and the significance of the wave I study are considered to be the same.

## 3. Discussion

### 3.1. Patterns of BAEP Wave Change during Microvascular Decompression for Hemifacial Spasm

#### 3.1.1. Latency and Amplitude of BAEP Wave V

When BAEPs are inspected using a real-time method, a waveform is formed within 10 s. Therefore, the change in the waveform can be observed every 10 s and the change in the waveform can be detected very precisely. In particular, the change in the latency and amplitude of wave V of BAEPs is observed in a very different pattern; the change in wave V latency is prolonged slowly and continuously. However, the wave V amplitude rapidly decreases by 50% within 10 s and, in some cases, wave loss occurs suddenly.

Looking at the process of forming a waveform while measuring BAEPs, the waveform is shaped like a skeleton before averaging 300 iterations. The shape of the waveform formed in this way does not change significantly, even if the averaging time advances up to 1000 times, and the shape continues to be maintained. In the case of testing with a stimulation rate of 10 Hz and an averaging time of 1000, the latency of wave V, which gradually and continuously changes, is reflected in the test waveform. However, the amplitude of wave V, which rapidly decreases within 10 s, is seldom reflected because hundreds of normal averaged waveforms already exist.

Therefore, if the BAEP waveform is tested in a way that cannot be observed within 10 s, the slowly progressive wave V latency may be discernable, but the rapidly changing amplitude will be difficult to detect [17].

#### 3.1.2. Time-Dependent Classification of BAEP Wave Changes

The change in the waveform can be divided into the case of rapid change and the case of showing slow change; this can be classified into three concepts [17].

Phase I: When the operation is stable, there is no change in the amplitude of the V wave and only the wave V latency is observed little by little in less than 1 ms.

In general, the change in the wave V latency is slowly and continuously extended for several minutes to several tens of minutes when the dura is opened, even if there is no particular abnormality. After the main procedure, the original waveform will gradually recover.

This stage corresponds to almost all situations of surgery, except for the main procedure, and it is the period which is observed for the longest time.

Phase II: This is a state in which changes in both the latency and amplitude of wave V are observed. When affected by a brain retractor or manipulation during this procedure, an extended wave V latency of more than 1 ms is observed. However, it can be observed that the wave V amplitude decreases by more than 50% within 10 s. When wave V is extended for more than 1 ms, the probability of decreasing the amplitude of wave V is greatly increased.

This stage occurs in the main procedure and during decompression of the offending vessels and facial nerves.

Phase III: When the brain retractor is used excessively in the main procedure, if the vestibulocochlear nerve is greatly affected, BAEP wave V loss occurs very quickly and suddenly.

This step occurs when the offending site is deep or excessive retraction is used in the main procedure. It often happens in as little as 10 s and, when this condition occurs, it is very dangerous.

In general, more serious damage occurs when all waveforms suddenly disappear after no waveform change compared with when wave V disappears after being delayed by 1 ms and reduced by 50% amplitude.

#### 3.1.3. Classification of BAEP Waves and Cochlear Nerve Damage

In the case of Phase I, where only the extension of wave V latency is observed very slowly and continuously, it accounts for more than half of all surgeries; this phenomenon is observed even with only dura open. Waveform changes due to traumatic mechanical damage are observed in Phases I, II, and III and can be divided into four stages (mild, moderate, severe, and critical damage) depending on the degree of direct damage to the vestibulocochlear nerve [17] [Table 4].

Mild damage: This is the case where no significant change in the waveform is observed, in which the latency of wave V is extended to less than 1 ms or the amplitude is decreased by less than 50%. In this case, waves I, III, and V are clearly observed. The state in which the latency of wave V is extended by more than 1 ms and the amplitude is reduced to less than 50% also applies. This corresponds to the Phase I stage.

Moderate damage: This is a more advanced damage stage from mild damage, in which the latency of wave V is extended by more than 1 ms and the amplitude is reduced by more than 50%. It is important that the amplitude is reduced by more than 50%. If the effect is not stopped by surgery, most of them will decrease by more than 80% or progress to wave V loss. This corresponds to the Phase I stage.

Severe damage can be classified into several types.

Severe damage (1): The wave V delay is greater than 2 ms and the wave V amplitude is reduced by more than 80%. When the wave V latency is delayed by more than 2 ms, the V wave amplitude often decreases rapidly. Therefore, even if the latency is delayed by more than 2 ms without the amplitude change in wave V, the surgeon must be notified. This corresponds to the Phase II stage.

Severe damage (2): This is a case where Phase II suddenly occurs without going through the mild and moderate nerve damage of Phase I. This is when BAEP wave abrupt change occurs. When vestibulocochlear nerve focal damage is severe, the amplitude suddenly decreases without changing the latency of wave V. This corresponds to the Phase II stage.

Severe damage (3): This is a change in the waveform due to vascular circulation damage. As with Phases I and II, the reproducibility of the waveform suddenly disappears without any change in the latency or amplitude of wave V.

If artifacts are mixed, the waveform shakes without reproducibility and then all wave loss occurs. 

If there is a change in the morphology of the BAEP waveform, it is because the vascular circulation is disturbed. In this case, a vasodilator such as papaverine should be administered to facilitate the vascular circulation of the surrounding blood vessels. Otherwise, if left unattended, all wave loss will be observed where wave I is not observed. After the operation, the patient will not only complain of overall disorders of the vestibular system but also complain of hearing impairment. This corresponds to the Phase II stage.

Critical damage: BAEP wave loss state. This is divided into traumatic mechanical damage in which wave I is observed and vascular circulation damage in which all wave loss is observed without wave I. BAEP wave loss can cause hearing loss after surgery. In particular, there are many cases that occur suddenly, such as severe damage (2) of Phase II. Therefore, it is important to observe the BAEP test continuously during surgery. This corresponds to the Phase III stage.

In most cases, a change in the wave V latency of the waveform is observed, followed by an observation of a sequential decrease in the wave V amplitude. A much more dangerous situation is when there is no change in the waveform and then a sudden change in latency or amplitude is observed.

#### 3.1.4. Practical BAEP Warning Criteria

In most cases, it is difficult to return the BAEP waveform to normal after considerable changes occur during surgery, meaning that hearing loss after surgery is possible. Various known existing warning criteria connect waveform changes and hearing status during and after surgery.

We should consider it important to notify in advance before a serious change in BAEP waveform occurs. From this point of view, the “prewarning sign” can help detect the condition of the auditory nerve during surgery in advance, meaning a decision can be made regarding whether to proceed with the surgery or to stop for a while. When there is no change in BAEP wave V amplitude, if prolongation occurs from wave I, the operation proceeds without interruption. If prolongation occurs from wave III, the severity is emphasized by being notified of the situation from prolongation of about 1 ms and the operation is stopped. Then, when the waveform is restored, the surgery is performed again.

The application of this prewarning sign helps considerably in reliably recovering the waveform after the BAEP waveform changes during surgery.

## 4. Conclusions

Postoperative hearing loss due to injury of the vestibulocochlear nerve is a complication during MVD for HFS. The incidence of this complication could be greatly reduced by intraoperative neurophysiological monitoring of BAEPs.

Advances in BAEP testing have provided neurosurgeons with greater insight during MVD surgery and may prevent vestibulocochlear nerve damage.

Future studies on the development of novel monitoring techniques and the modification and optimization of existing BAEP methodologies will continue to improve clinical outcomes in terms of hearing changes following MVD for HFS.

## Figures and Tables

**Table 1:** BAEP groups of change patterns.

Group	Latency	Amplitude	N = 463	Post OP HL
1	≤1 ms	≤50%	346	0
2	≥2 ms	≤50%	38	0
3	≤1 ms	≥50%	15	0
4	≥2 ms	≥50%	36	1
5	Permanent loss		5	5
6	Transient loss		23	0

BAEP wave V standard (data from [17]).

**Table 2:** Proportion of patients according to the final change in wave V in the group prolonged from wave I and the group prolonged from wave III.

The Final Change in Wave V	Group A Prolonged from Wave I	Group B Prolonged from Wave III	*p*-Value
Latency prolongation under 1 ms with amplitude decrement under 50%	72 (88.9%)	105 (65.6%)	<0.001
Only latency prolongation of 1 ms or over	8 (9.9%)	45 (28.1%)	0.001
Only amplitude decrement greater than 50%	0	0	ns
Latency prolongation over 1 ms with amplitude decrement over 50%	0	0	ns
Wave V loss	1 (1.2%)	10 (6.3%)	0.078
Total	81	160	

Analysis of 241 patients with latency prolongation over 1 ms or amplitude decrement over 50% in 1025 patients (data from [18]).

**Table 3:** The rate of complications according to the presence or absence of wave I in patients with BAEPs wave V loss.

	Persistence of Wave I	Absence of Wave I	*p* Value
Patients, n	24	12	
Hearing loss, n (%)	2 (8.33%)	6 (50.00%)	0.009
Subtype of hearing loss, n (low/high/total)	2:0:0	0:0:6	
Dizziness, n (%)	0	5 (41.67%)	0.002
Tinnitus, n (%)	0	3 (25.00%)	0.031
Diplopia, n (%)	0	1 (8.34%)	0.333
Hoarseness, n (%)	0	1 (8.34%)	0.333

Analysis of 36 patients with BAEPs wave V loss in 670 patients. HL—hearing loss; low —low-frequency hearing loss, high—high-frequency hearing loss; total—total hearing loss (data from [20]).

**Table 4:** The relationship between the phase and the degree of nerve damage is explained by the change in the waveforms.

	Damage Degree	Latency Change		Amplitude Change
Phase I	Mild	≤1 ms		≤50%
Phase II	Moderate	≥1 ms		≥50%
	Severe	1	≥2 ms	≥80%
		2	No change	≥50% (abrupt change)
		3	No reproducibility	
Phase III	Critical	1 2	Loss, traumatic mechanical damage Loss, vascular circulation damage	

(Data from [17]).

## Data Availability

All data generated or analyzed during this study are included in this published article.

## Abbreviations

BAEPs—brainstem auditory evoked potentials; MVD—microvascular decompression; HFS—hemifacial spasm; nHL—normal hearing level; SPL—sound pressure level; HL—hearing loss.
